# Distance- and Angle-Based Hybrid Localization Integrated in the IEEE 802.15.4 TSCH Communication Protocol

**DOI:** 10.3390/s24123925

**Published:** 2024-06-17

**Authors:** Grega Morano, Aleš Simončič, Teodora Kocevska, Tomaž Javornik, Andrej Hrovat

**Affiliations:** 1 Jožef Stefan Institute, Jamova cesta 39, 1000 Ljubljana, Slovenia; ales.simoncic@ijs.si (A.S.); teodora.kocevska@ijs.si (T.K.); tomaz.javornik@ijs.si (T.J.); andrej.hrovat@ijs.si (A.H.); 2 Jožef Stefan International Postgraduate School, Jamova cesta 39, 1000 Ljubljana, Slovenia

**Keywords:** integrated localization and communication (ILAC), hybrid localization, direction of arrival (DoA), multi-carrier phase difference (MCPD), IEEE 802.15.4, time-slotted channel hopping (TSCH), AT86RF215

## Abstract

Accurate localization of devices within Internet of Things (IoT) networks is driven by the emergence of novel applications that require context awareness to improve operational efficiency, resource management, automation, and safety in industry and smart cities. With the Integrated Localization and Communication (ILAC) functionality, IoT devices can simultaneously exchange data and determine their position in space, resulting in maximized resource utilization with reduced deployment and operational costs. Localization capability in challenging scenarios, including harsh environments with complex geometry and obstacles, can be provided with robust, reliable, and energy-efficient communication protocols able to combat impairments caused by interference and multipath, such as the IEEE 802.15.4 Time-Slotted Channel Hopping (TSCH) protocol. This paper presents an enhancement of the TSCH protocol that integrates localization functionality along with communication, improving the protocol’s operational capabilities and setting a baseline for monitoring, automation, and interaction within IoT setups in physical environments. A novel approach is proposed to incorporate a hybrid localization by integrating Direction of Arrival (DoA) estimation and Multi-Carrier Phase Difference (MCPD) ranging methods for providing DoA and distance estimates with each transmitted packet. With the proposed enhancement, a single node can determine the location of its neighboring nodes without significantly affecting the reliability of communication and the efficiency of the network. The feasibility and effectiveness of the proposed approach are validated in a real scenario in an office building using low-cost proprietary devices, and the software incorporating the solution is provided. The experimental evaluation results show that a node positioned in the center of the room successfully estimates both the DoA and the distance to each neighboring node. The proposed hybrid localization algorithm demonstrates an accuracy of a few tens of centimeters in a two-dimensional space.

## 1. Introduction

The emergence of context-aware applications in industry and smart cities requires localization functionalities in Internet of Things (IoT) wireless communications to improve operational efficiency, safety, user experience, and resource management. In industrial applications, localization is crucial for tracking assets, managing inventory efficiently, and optimizing logistics by precisely locating equipment and goods in real time [1,2]. Smart cities benefit from localization through improved public safety and traffic management systems, where the precise location of vehicles and pedestrians can significantly improve routing efficiency and emergency response [3]. In smart homes, localization functions enable more sophisticated control and automation, adjusting lighting, heating, or security systems based on the presence and movements of occupants. In consumer applications, such as indoor navigation in museums, malls, or airports, localization can significantly improve the user experience by providing more accurate and reliable directions [4]. Localization with wireless systems can be achieved by observing the communication channel’s characteristics during packet exchange; thus, the same hardware can be used for dual purposes [5]. Integrated Localization and Communication (ILAC) [6,7] enables IoT devices to simultaneously exchange data and determine and report their position in space, maximizing resource utilization while reducing deployment and operation costs.

In challenging scenarios with many obstacles and complex propagation, IoT protocols that can mitigate interference and multipath fading and ensure that consistent data transmission are promising enough to provide localization functionality. IEEE 802.15.4 Time-Slotted Channel Hopping (TSCH) [8] is particularly suitable due to its use of the channel hopping technique, which can efficiently mitigate the issues of interference and multipath fading by changing the transmission frequency in a pseudo-random manner [9]. Low power consumption and high reliability make the protocol suitable for critical IoT applications in harsh environments. Integrating accurate localization functionality into TSCH will further enhance the effectiveness and operational capabilities of the protocol and open up new possibilities for automation, monitoring, and interaction within physical environments. The integration design has to ensure that communication reliability and network efficiency are not adversely affected by the additional localization capabilities.

Localization based on channel metrics from wireless systems, where the location of a target device is determined based on its interaction with devices with known locations referred to as anchors, has been used for indoor environments where satellite-based localization systems optimized for outdoor environments do not operate [1,10]. The location can be determined with the fingerprinting technique or by using more advanced and accurate geometric methods based on a separate or joint use of distance and angle estimations. Lateration is a common two-dimensional (2D) localization method that uses distance estimation from the target to at least three anchor devices. The distance between devices can be estimated through various techniques that include Received Signal Strength Indicator (RSSI), Time of Arrival (ToA) and Phase-Based Ranging (PBR). While RSSI-based methods estimate distance by measuring signal attenuation, they often suffer from significant inaccuracies due to multipath fading, interference, and varying environmental conditions [11]. ToA-based methods, which calculate the distance by measuring the travel time of signals, also face challenges, such as the need for precise synchronization and the impact of signal reflections. PBR can provide more accurate distance estimation than techniques based on RSSI and ToA, even in rich multipath environments [12]. A technique called Multi-Carrier Phase Difference (MCPD) ranging uses the phase difference of a received signal at multiple frequencies to estimate the distance with high precision [13].

Another common 2D localization method is angulation, which uses the angle measurements related to the direction of the received or transmitted signals [14]. Direction of Arrival (DoA) estimation with rotary antennas involves using a rotating directional antenna to measure signal strength at different angles, identifying the direction from which the signal is strongest or most consistent [15]. However, rotary antennas are impractical to implement due to their mechanical complexity and slow response time. Instead, phase interferometry is often employed, where multiple antennas spaced by a distance shorter than half of a signal’s wavelength are formed into an antenna array. As a signal phase propagates in space, its variations in the phase measured across antenna elements of the array can be used to estimate the direction with high-resolution DoA algorithms, such as MUltiple SIgnal Classification (MUSIC) or the Estimation of Signal Parameters via Rotational Invariance Technique (ESPRIT) [14]. Recent advancements in DoA estimation explore full-hardware approaches that reduce the computational overhead of classical algorithms and offer angle estimation response in real time [16].

The hybrid localization method combines the lateration and angulation methods, where the 2D location of a target device can be estimated with only a single anchor node, which is beneficial for systems with a single receiver or access point. Even if the goal is not to minimize the number of anchor devices, the hybrid localization method can be beneficial, as fusing measurements from multiple anchors can greatly improve the accuracy of the localization system [1]. A straightforward approach to achieve hybrid localization is to combine the ToA and DoA estimates. Wi-Fi networks offer great opportunities for hybrid localization because they provide Channel State Information (CSI) that can be extracted alongside communication. CSI contains the amplitude and phase information of the signal transmitted at multiple subcarriers between different antenna elements, which can be used to extract both DoA and ToA estimates [17,18]. Several hybrid localization solutions have been proposed for ultra-wideband (UWB) technology. When an antenna array is employed at the receiver, the 2D MUSIC algorithm can be used for joint estimation of DoA and ToA [19].

An efficient approach for integrating localization functionality in IEEE 802.15.4 TSCH networks has not yet been proposed in the literature. The level of accuracy required for current and future IoT applications cannot be achieved with the available localization techniques that rely on either RSSI [20] or ToA-based [21] lateration. The proposed modifications in solutions using PBR for lateration [22] disrupt the normal operation of the communication process and lead to increased complexity and reduced network performance. Angulation and hybrid localization techniques have not yet been considered for implementation in the protocol. In this study, we propose a method for integrating hybrid localization into the IEEE 802.15.4 TSCH protocol, designed to minimize disruptions to the communication network. The approach ensures that devices can perform localization alongside communication without requiring any special network deployment changes. Our solution builds on previous research, where we developed methods for integrating MCPD distance estimation [23] and DoA estimation [24] into the TSCH protocol. We synthesize findings and insights obtained to introduce a comprehensive hybrid localization solution within the TSCH protocol. We discuss the requirements necessary for successful angle and distance estimation and introduce a scheme that operates concurrently with communication. With the proposed enhancement, a node can transmit a packet to a target device and estimate both the DoA and the distance to the receiving node, which can be used for localization. We validate our proposed localization method in a real-world setting using low-cost proprietary devices. The main contributions of the paper are as follows:

- Proposing a novel approach for hybrid localization based on distance and angle that is integrated alongside TSCH communication.
- Demonstrating the feasibility and effectiveness of our approach in a real-world scenario using low-cost proprietary devices.
- Providing software incorporating our approach that can be of service in future research.

The remainder of this paper is organized as follows. The integration of localization functionality alongside communication in various IoT wireless technologies is reviewed in Section 2. Section 3 discusses the requirements for localization with the TSCH protocol and provides a theoretical background on distance and DoA estimation. The proposed modification of the TSCH protocol is presented in Section 4. The focus in Section 5 is on describing the details of our proprietary system. Section 6 presents and discusses the experimental evaluation results. Section 7 concludes the paper and outlines future work.

## 2. Related Work

Acquiring location estimation alongside communication has become an extremely useful and desired feature of wireless networks. Various wireless technologies have recognized the need for precise localization and have started integrating localization techniques into the protocol specifications, as summarized in Table 1. This section overviews the recent advancements in ILAC for the most commonly used technologies in IoT setups.

The Wi-Fi communication technology specified in IEEE 802.11 is widely adopted for high-speed data transmission in various IoT applications when power consumption is not a limiting factor within the system. Wi-Fi technology is increasingly utilized for indoor localization solutions mainly based on CSI [25]. The detailed information about channel conditions between the transmitter and receiver, including data on amplitude and phase for each subcarrier, makes CSI-based localization a cost-effective solution for indoor positioning systems [26]. Machine learning (ML) approaches have been proven to be effective for fingerprinting-based localization in scenarios where a large amount of channel measurement data is available for building the ML models [27]. Recognizing the need for wireless localization, the IEEE 802.11az-2022 amendment greatly enhanced localization performance by leveraging the mmWave spectrum and dedicated beamforming techniques [28]. The Wi-Fi standard continues to evolve to incorporate enhanced sensing capabilities with Integrated Sensing and Communication (ISAC) aspects through the development of the IEEE 802.11bf amendment [29].

Providing accurate location and timing information was within the focus of UWB technology for low data rate communication, introduced in 2007 by standard IEEE 802.15.4a [30]. Short-duration pulses and wide bandwidth provide good temporal resolution for accurate ToA and Channel Impulse Response (CIR) estimates [31,32]. The IEEE 802.15.4z UWB physical (PHY) enhancements, released in 2020, include additional improvements for higher accuracy as well as secure localization and ranging in multipath environments [33,34]. Recent research has focused on enhancing UWB-based localization systems by integrating advanced signal processing algorithms and ML techniques [35].

Bluetooth Low Energy (BLE) is widely adopted in IoT devices due to its low power consumption, high data rate connectivity, and compatibility with smartphones and wearables. BLE supports basic localization functionalities using RSSI measurements, allowing fingerprint-based and proximity-based localization [36,37]. A significant advance in BLE’s capabilities for localization services was introduced with the release of Bluetooth 5.1, which enables devices to estimate the DoA and determine the angle from which a signal arrives at a receiver [38,39]. Recent developments in BLE standardization aim to further enhance the accuracy of localization by adding the channel sounding feature alongside the communication protocol [12]. This feature uses PBR to analyze the phase information of the radio signal and thus enables precise distance estimation between devices, even in challenging multipath environments.

Conversely, for IoT scenarios that require low power and low data rate connectivity with a potentially large number of devices in a relatively large area, various alternative IoT protocols are employed, such as ZigBee, Thread, 6LoWPAN, and Wireless HART. These protocols are based on the IEEE 802.15.4 standard and its TSCH protocol, added in the 2016 amendment [8]. There have been several studies on applying the IEEE 802.15.4 standard and related IoT technologies for localization, primarily using the RSSI metric for proximity-based and fingerprint-based localization methods [20,40]. Although the accuracy of RSSI-based localization systems is continuously improving with the development of ML algorithms [41], the main drawback remains. The high sensitivity of the RSSI metric to environmental changes [11] makes the localization system unreliable and case-specific to the particular scenario. Some IEEE 802.15.4 compliant radios available on the market provide a feature that triggers an interrupt signal at the start of the detected data packet. This ability was exploited for ToA-based ranging that is compatible with the IEEE 802.15.4 standard, achieving distance estimation accuracy at a range of several meters [21,42].

Recent advancements in radio design have introduced the option of measuring the phase of received signals, motivating research into PBR. With the use of the MCPD method [43], also referred to as Active Reflector (AR) [22], the presented solutions achieved even greater accuracy in ranging and localization compared to other methods [22,43,44]. Nevertheless, there has been little consideration of how the PBR process affects the overall communication process. Communication is often interrupted or delayed for longer periods to allow devices to measure the signal’s phase. Additionally, these solutions focus on specific use-case scenarios, where nodes are used only for localization and do not adequately cover the more typical network deployments destined for communication.

**Table 1:** Localization techniques for different wireless technologies.

Technology	Communication				Localization		
	Range	Throughput	Power Usage	Radio Signal	Metric	Advantages	Disadvantages
Wi-Fi [29]	15–100 m	54 Mbps– 9.8 Gbps	High	OFDM	RSSI [26,27]	Simple to implement; Widely available	Prone to multipath effects, noise, and environmental changes; Low accuracy
					CSI [25,26,45]	More robust to noise and multipath; High accuracy	Not widely available on of-the-shelf devices; Complex processing algorithms
UWB [46]	10– 100 m	6.8– 460 Mbps	Low	PPM	ToA (TDoA) [31,33,47]	Immune to interference; Less susceptible to multipath effects; High accuracy	Shorter range; Requires wide bandwidth; Need for synchronization between multiple anchors
BLE 5.1 [48]	10–200 m	125–2000 kbps	Low	GFSK	RSSI [36,37]	Simple to implement; Readily available on consumer devices	Prone to multipath effects, noise, and environmental changes; Low accuracy
					DoA [38,39]	High accuracy; Less susceptible to multipath effects; Requires fewer anchors for localization	Requires switch antenna array; Complex processing algorithms for higher resolution
					PBR [12,13]	High accuracy; Less susceptible to multipath effects	Complex processing algorithms; Longer time for sampling at multiple frequencies
IEEE 802.15.4 [8]	100 m	250 kbps	Low	O-QPSK	RSSI [20,40]	Simple to implement; Metrics available alongside communication	Prone to multipath effects, noise, and environmental changes; Low accuracy
Proposed					PBR, DoA	High accuracy; Less susceptible to multipath effects; Single anchor node required for localization	Requires switch antenna array; Longer time for sampling at multiple frequencies; Complex processing algorithms

## 3. Localization in TSCH

**Figure 1:**
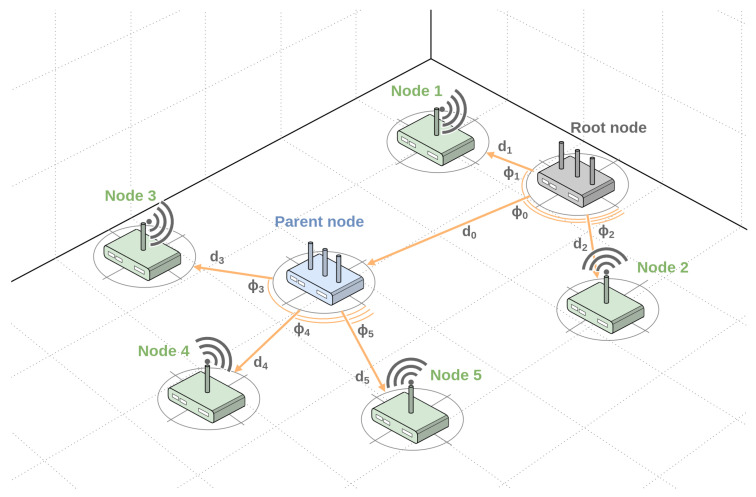
Topology of the destination-oriented directed acyclic graph (DODAG) network with the proposed method of hybrid localization, where a single node referred to as the root/parent node can determine the location of its child nodes.

In TSCH networks, the Routing Protocol for Low-Power and Lossy Networks (RPL) is usually deployed, which creates a destination-oriented directed acyclic graph (DODAG) topology [9], as shown in Figure 1. In this configuration, one node is a dedicated DODAG root, while others are parent and child nodes. The packets in the network generally traverse from the child nodes to the parent nodes and eventually reach the root node, which then forwards them down to the destination device. This poses a challenge for localization because any two arbitrary devices cannot exchange messages directly in the default configuration and, therefore, cannot effectively measure the characteristics of the communication channel between them. Therefore, integrating lateration or angulation methods into the TSCH protocol is difficult to realize without extensive communication protocol and network structure changes. One of the possible solutions is the use of hybrid localization with a single node, where the root or parent node undertakes the localization of other devices. The root/parent node can address its child nodes arbitrarily, send packets to them, and measure the properties of the communication link. To implement hybrid localization with a single node, both the angle of arrival and the distance to the device of interest are required.

### 3.1. Estimation of Direction of Arrival

The phase difference in the incoming signal at multiple antennas, separated by a distance shorter than half the wavelength of the signal, can be used to estimate the direction from which a signal is received [24]. High-resolution algorithms can be applied at the receiver to analyze how the phase of the signal changes across the antenna array and to determine the angle at which the signal arrives [49]. In this study, we validated the effectiveness of the MUSIC algorithm, which is considered adequate for real scenarios due to its resilience to noise.

The phase measurement for different antennas in systems with a single radio frequency (RF) chain, such as the radios that support IEEE 802.15.4, is commonly approached with an antenna switching circuit. It allows the device to switch between antennas and sample the phase with various switching patterns, providing application-oriented features. In this study, the Round Robin switching pattern was used, as it provides the most phase samples per antenna, which benefits the MUSIC algorithm. Although switching antenna arrays are suitable for developing a cost-effective and energy-efficient solution, they introduce a time delay between the measurements from each antenna, which can lead to large errors in the case where the Carrier Frequency Offset (CFO) between the devices is not compensated.

### 3.2. Estimation of Distance between Two Devices

A distance between devices can be determined using phase differences across multiple frequencies, which can be measured using the MCPD method. In this method, one of the devices acts as the initiator, and the other acts as the reflector. To eliminate initial unknown offsets in the phase of the generated signal, the devices alternately measure the phases: first, the initiator transmits a continuous wave (CW), and the reflector measures the phase of the received signal, whereupon the roles are reversed. To increase the maximum unambiguous range (UR) and spatial resolution, the devices extend the apparent bandwidth by repeating the phase measurements at several frequencies and later construct the phase response from them. The frequency step between the frequencies determines the UR, while the total bandwidth used determines the resolution of the system.

The method of obtaining phase samples can be either uniform [22] or non-uniform [50], and it can include weighting of the phase samples [51]. After acquiring the phase response of a link, spectral analysis techniques can be employed to estimate the distance between the devices. Among the various algorithms available, such as slope-based [52], Fast Fourier Transform (FFT)-based [53], MUSIC-based [43], and ML-based [54], we use the FFT-based interpolated Complex-valued Distance Estimation (iCDE) algorithm [55] in this study due to its robustness to noise and ability to mitigate multipath effects.

## 4. Integration of Localization Functionality

Integrating localization functions into the TSCH protocol requires a thorough analysis of DoA and MCPD estimation requirements for effective implementation. A novel comprehensive hybrid localization solution using the TSCH protocol is developed considering the findings from our proposed methods for TSCH protocol enhancement with MCPD distance estimation [23] and DoA estimation [24]. The approach was selected to evaluate the hybrid localization performance by solely modifying the PHY and Medium Access Control (MAC) layers of the IEEE 802.15.4 standard. Although altering the upper layers of the communication protocol might present numerous potential enhancements and performance optimization, these are beyond the scope of this study.

### 4.1. Design Considerations

The main objective of the proposed localization process is to minimize its impact on communication while ensuring that the required changes in the communication protocol remain minimal. A short interruption of the communication process is introduced for the devices to measure the communication channel characteristics in a Phase Measurement Process (PMP) procedure. A single angle and distance to the neighbor estimate are obtained to minimize the impact on communication, although multiple estimates are feasible.

**Figure 2:**
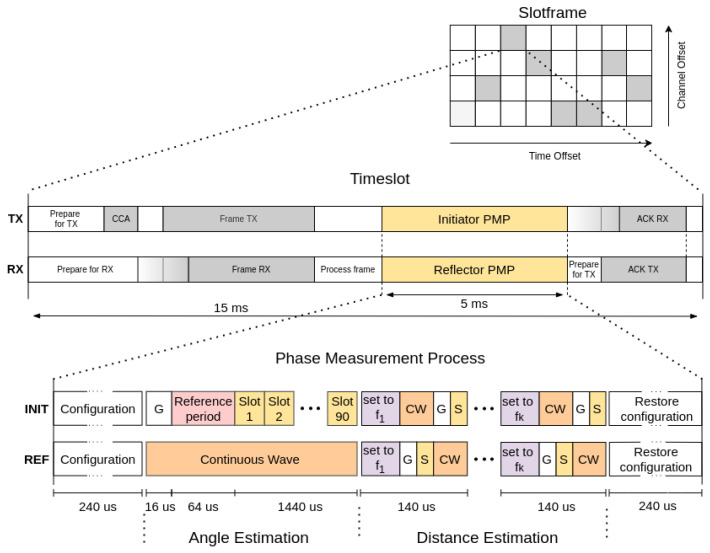
Integration of a phase measurement process into the Time Slotted Channel Hopping (TSCH) timeslot.

Simultaneous initiation of the measurement is needed for an appropriate channel estimation. The globally synchronized network inherently provided by the TSCH protocol is used to satisfy the synchronization requirement. The protocol utilizes a slotframe in which the communication time is divided into timeslots that coordinate the intervals for the preparation of packets, the reception/transmission of user data, and the exchange of acknowledgment. To add localization functionality to the protocol, we propose to extend the timeslot with an additional interval that allocates the required time for PMP, as shown in Figure 2. The newly defined TSCH timeslot timings enable both devices to simultaneously start with the channel measurement. Leveraging the TSCH protocol further, PMP is positioned immediately following the data packet exchange, allowing the measured phase information to be returned to the initiator as an information element within the acknowledgment exchange.

For DoA estimation, the PMP allocates 90 slots where a device can select the desired antenna element and sample the phase of the signal. This allocation is determined as a trade-off between the sensing time and the communication time to provide sufficient samples per antenna, enabling various switching patterns with different numbers of antenna elements [24]. Since a single RX chain with an RF switch is used to capture phases from different antennas, the phase measurement accuracy decreases with the increasing sampling time interval due to the CFO. The cumulative phase offsets induced by the CFO significantly affect the accuracy of the DoA estimation. A reference period is added to the PMP to address this, where eight samples can be obtained from a single antenna. The reference period can be used for CFO estimation so that calibration can be used to compensate for the phase offsets [24], enhancing the overall accuracy of the DoA estimates.

To estimate distance with MPCD, a sequential phase sampling through the available RF spectrum is required. Although measuring the phase for each communication channel during packet transmission and then combining the measured phases into phase response is theoretically feasible, practical challenges arise due to the significant channel variations between two consecutive packet transmissions. In addition, the time between phase measurements at the initiator and reflector should be minimized to achieve accurate distance estimation [13]. We propose executing the entire RF spectrum sweep sequentially within the shortest possible interval. To minimize the measurement time and avoid communication interruptions, a Golomb set of frequencies is used [50]. With this approach, only 15 frequencies are needed for a complete phase response reconstruction, significantly reducing the time compared to other methods that might require up to 160 samples [22]. This is beneficial for introducing localization into communication, as more time can be spent on communication than sensing. The reduced number of frequencies leads to a lower probability of interference with other wireless technologies and more efficient spectrum use. After expanding the measured data [23], the devices can estimate the range with the UR up to 300 m.

In TSCH networks, the communication between a parent node and its neighbor is orchestrated through a schedule known as a slotframe, where each packet exchange occurs in a designated cell. Since the location estimate is obtained with each transmitted packet, the location estimation updates occur at the same rate as packet exchanges. The parent orchestrates multiple devices, and the actual update rate depends on the number of child nodes that the parent has to communicate with. If a new node joins the network, a cell is added to the slotframe, reducing the update rate for all devices. The slotframe is managed by a Scheduling Function (SF), which is not defined in the IEEE 802.15.4 TSCH standard. Nonetheless, the flexibility of the slotframe provides numerous opportunities for improvements in the performance and scalability of the localization system. In case a device requires a faster update rate, more cells can be added to the slotframe for a packet exchange with this device. If a node is recognized as static and does not change its position, the cells can be removed, freeing up space in the slotframe for other devices. The responsiveness and accuracy of the system can be optimized according to the network requirements using a dedicated SF in comprehensive applications.

### 4.2. Phase Measurement Process

The structure of PMP includes steps for: (1) configuring the radio for phase measurement, (2) estimating the angle, (3) estimating the distance, and (4) restoring the configuration of the radio to continue with the communication. The proposed generic solution, which is applicable to any analog radio, has been modified for the currently available, low-cost, off-the-shelf radios that offer a phase sampling rate of 8 μs [24]. The intervals shown in Figure 2 were selected accordingly, and the timeslot was extended from the default 10 ms to 15 ms.

During the angle estimation, the reflector initiates a CW transmission on the same frequency channel that was used to transmit the data packet. The initiator first waits for a predefined guard period denoted by G in Figure 2 and measures 8 phase samples on the reference antenna for CFO calibration. Then, 90 slots are available for phase measurement with different antenna elements according to the selected switching pattern. To mitigate the phase distortion introduced by altering the signal’s path during the measurement, enough time is provided in each slot to sample the phase and to switch to the desired antenna element.

After estimating the angle, the devices start estimating the distance with the MCPD technique. Both devices configure their Phase-Locked Loop (PLL) to a predefined frequency. The initiator then transmits CW, waits for a predefined guard time, and performs a phase sampling denoted as S in Figure 2, while the reflector waits for a guard time interval, samples the phase, and later initiates CW transmission. The process is then repeated at different frequencies until all frequencies in the set are used. The defined PMP scheme with exact intervals enables periodic timer interrupts that ensure the devices remain synchronized, which is crucial for accurate distance estimation.

## 5. Experimental Setup

**Figure 3:**
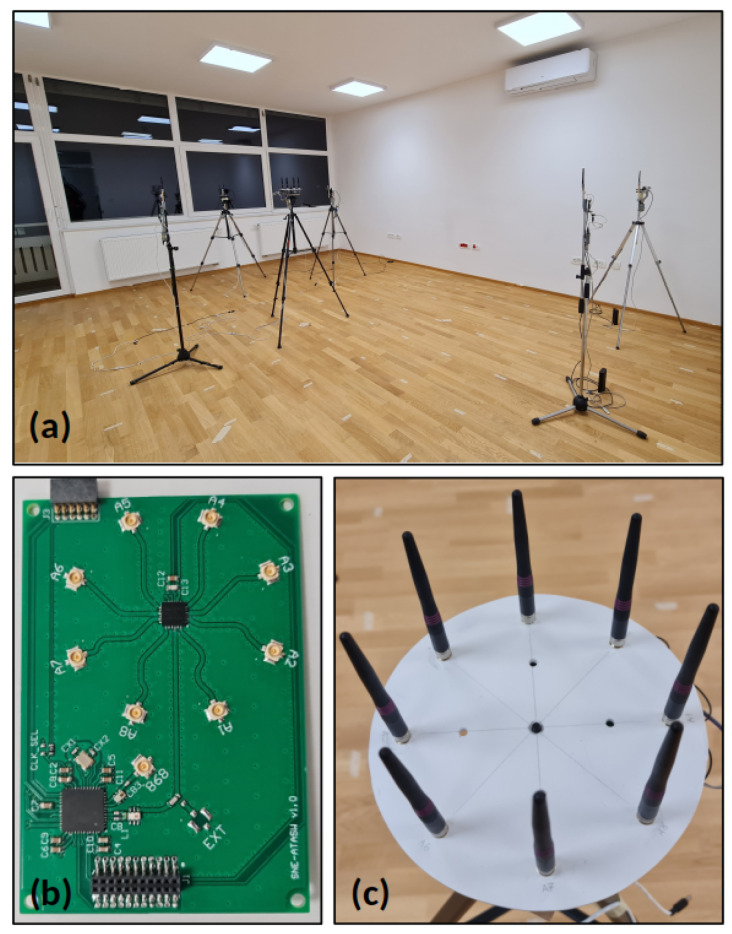
(**a**) Experiment setup. (**b**) Micro-controller equipped with AT86RF215 radio and RF switch. (**c**) Uniform circular antenna array with eight elements.

To evaluate the performance of the proposed localization in real environments, a test setup shown in Figure 3 was created in an office with low-cost micro-controllers [56]. The IEEE 802.15.4 TSCH network is formed with six devices, and the root node attempts to determine the position of the other five nodes during the communication.

### 5.1. System Design

The micro-controller devices are built upon the AT86RF215 [57], an IEEE 802.15.4 compliant radio with an option for register-based monitoring of the received signal’s phase through the Phase Measurement Unit (PMU). Furthermore, the radio can measure the In-Phase and Quadrature (IQ) components of the received signal, the phase stability or Quality Factor (QF), and the frequency offset [57]. With additional configuration options, the PMU can sample the aforementioned data with a period of 8 μs, which can be accessed via a Serial Peripheral Interface (SPI).

To expand the single RF signal chain of the radio, the platform incorporates an RF switch that can accommodate an antenna array with up to eight elements. The board design ensures that all traces from the RF switch to the antenna connectors are of equal length and that each path contributes equally to the overall phase delay without causing errors in DoA estimation. The co-planar waveguide with a via fence was applied to reduce the cross-talk between the traces. A Uniform Circular Array (UCA) consisting of eight dipole antennas was used for localization, as depicted in Figure 3c. UCA offers an advantage over linear arrays, as it can estimate angles from 0° to 360°. The chosen number of antenna elements is considered as a trade-off between the physical size of the device and its localization performance.

### 5.2. Radio Configuration

During the normal operation of the radio, the internal baseband core generates and receives the RF signals. The phase of the signal can be measured only after the radio has been configured adequately. A brief description of its configuration for phase measurement is provided for completeness. Additionally, a driver for the AT86RF215 radio within the Contiki-NG operating system [58] has been designed and developed. The driver is available online for future research [59].

The transceiver architecture works as a heterodyne receiver and direct up-conversion transmitter. The received signal is first converted to the intermediate frequency (IF), which can be 0.25 MHz, 0.5 MHz, 1 MHz, or 2 MHz. A band-pass filter with three possible cut-off frequencies can be selected for each IF through the register bits *RFn_RXBWC.BW*. The experimental analysis showed optimal phase measurement performance at an IF of 1 MHz. The initiator and reflector devices must be configured to measure the channel at the same frequency for accurate phase extraction. Since the receiver operates according to the heterodyne architecture, the transmitter must emit a CW at an IF frequency higher than the receiver is listening to. And since the PLL frequency should not be altered when the roles are reversed, the reflector must be configured to use inverted IF by setting the bit *RFn_RXBWC.IFI* to 1. According to the IF configuration, the receiver’s sampling frequency is set through *RFn_RXDFE* and must match the IF for optimal operation. The same registers configure the digital post filter for attenuation of out-of-band signals. The gain of the signal must remain the same during phase measurement to avoid phase changes. Thus, the bit *RFn_AGCC.EN* is set to 0 to enable manual control, and the receiver’s gain is set to a constant value through the register *RFn_AGCS*. In addition, the energy detection feature is disabled by setting the bit *RFn_EDC* to 0x03.

The front end of the transmitter is configured with *RFn_PAC* to set the output power, *RFn_TXCUTC* to configure the low-pass cut-off frequency and the ramp-up time of the power amplifier, and *RFn_TXDFE* to configure the digital front end of the transmitter. For *RFn_PAC*, the maximum value is typically selected, while for *RFn_TXCUTC* and *RFn_TXDFE*, the default values can be considered. To enable the transmission of CW, the radio is configured to use the digital-to-analog converter (DAC) overwrite feature, generating a local oscillator carrier signal at the selected frequency. By setting the register *RFn_TXDACI* to 0xFE and register *RFn_TXDACQ* to 0xBE, the DAC is overwritten with the maximum I and minimum Q signal magnitude. CW transmission is initiated by disabling the radio’s baseband with *RFn_IQIFC1.CHPM* set to 1 and issuing a command to transition the radio into a TX state. When measuring the phase, however, the baseband must be enabled (bit *RFn_IQIFC1.CHPM* set to 0), and the radio must be in the RX state. The PMU must also be configured and enabled. The PMU offers various configuration options: (i) to force the carrier frequency changes to occur at the end of the PMU period if *BBCn_PMUC.CCFTS* set to 1, (ii) to average the measured phase over the entire PMU period using the *BBCn_PMUC.AVG*), and (iii) the phase can be returned with or without normalization using the *BBCn_PMUC.IQSEL*.

### 5.3. Measurement Scenario

The measurements were performed in an empty office environment with dimensions 5.1 m by 6.5 m. During the measurement campaign, 17 Wi-Fi access points were in operation, and no special consideration was given to the possible interference. The measurement setup shown in Figure 3 includes six devices placed on a stand 1.4 m above the floor. The root node was positioned in the center of the room, with the other devices around for the root node to be a parent for all devices. After initiating the experiment, the root waited for all devices to join the network before transmitting packets to each device at regular intervals. With each received response, the root node forwarded the measured phases to a computer, which calculated the DoA and distance estimates. This simple method was chosen to test localization performance. However, it also offers numerous opportunities for improvement, such as distinguishing between static and mobile nodes, introducing burst packets for mobile nodes for a faster update rate, and dynamically assigning frequency channels for erroneous measurements.

## 6. Experiment Results and Analysis

The reference point for the estimated angle is positioned in the center of the UCA, while for the distance estimate, the reference point of the measurement is positioned at the first antenna element. For effective localization, this system error is corrected by converting the measured distance to the center of the UCA using the antenna radius and the angle at which the device is positioned. Furthermore, each distance measurement result contains an additional offset due to the radio signal paths on the circuit board and the antenna. In our case, we measured an offset of 2.05 m, and all measurement results were corrected accordingly.

The root node calculates the DoA and the distance to the target node after the transmission of each packet, regardless of whether the phase of the signal was measured accurately. Consequently, the estimations may be incorrect, which is often caused by channel interference or the loss of timeslot synchronization between devices in the network. We used the Quality Indicator (QI) value to identify and eliminate potential outliers. Both the iCDE and MUSIC algorithms generate a pseudo-spectrum as an output function. The peaks in the spectrum indicate the possible distance or angle estimates, and the magnitude of the selected peak represents the QI. The corresponding angle or distance measurements are discarded if the QI value falls below a predefined threshold. The total number of transmitted packets and the number of removed measurements are presented in Table 2.

However, detecting outliers based on the QI value can be unreliable in environments rich in multipath interference, such as the office environment. Different paths can introduce different phase shifts, resulting in constructive or destructive interference at the receiver. This is particularly evident in DoA estimation, where the device sometimes returns the DoA of the multipath component instead of the line-of-sight path of our interest [24]. The results from multiple frequencies can be used to mitigate the adverse effects of multipath interference, as different frequencies are not equally affected by the multipath effects. Given that devices in the modified TSCH perform DoA estimation at the same frequency at which the packet is transmitted, we can leverage this channel hopping feature. By aggregating the estimated data from multiple successive packets and calculating the median of these measurements, we can effectively filter out measurement outliers, minimizing the impact of multipath variations. In distance estimation processes, multiple frequencies are already employed within each measurement. Since we only sample the phase on 15 frequencies, the resulting phase response is vulnerable to noise. Increasing the number of sampled frequencies would enhance resilience to noise, but this would extend the time needed for obtaining measurements, potentially disrupting ongoing communications. Thus, taking the median of successive distance measurements will also enhance distance estimation accuracy by mitigating the multipath effects and noise.

**Figure 4:**
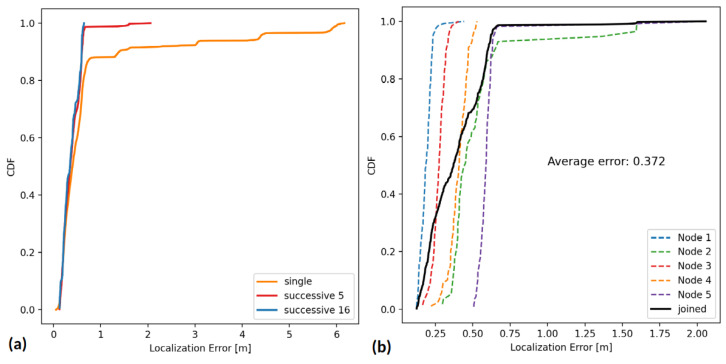
Cumulative Distribution Function (CDF) for estimated location errors. (**a**) Comparison of CDF with a single measurement with 5 and 16 successive measurements. (**b**) CDF for each node’s location with 5 successive measurements.

In the analysis, we considered a single measurement or a median of either 5 or 16 successive measurements as a final result. The effectiveness of location estimates is assessed with Root Mean Square Error (RMSE), which measures the average magnitude of the error between the estimated locations and the true locations. The RMSE gives a clear indication of the accuracy of localization system, but it is highly sensitive to large errors; thus, the Mean Absolute Error (MAE) is also presented, which is less sensitive to outliers and provides a direct average measure of error magnitude. The comparison of localization errors with different numbers of successive measurements is presented in Table 3. Figure 4a shows the comparison of the Cumulative Distribution Function (CDF). It provides insights into the accuracy of the localization system and visually demonstrates the point at which half of the estimated locations fall below the average error, which in all three cases is below 0.41 m.

When only a single measurement is considered as the final result, the maximum error exceeds 6 m. By adopting the median of 16 consecutive measurements to cover all available frequency channels, the maximum error drops to below 0.65 m. The proposed localization system achieves high accuracy using only a single node with a known location, compared to other techniques that require at least three anchors [40]. Acquiring many measurements is time-consuming for mobile device scenarios where a high update rate is essential for accurate device tracking. To quantify the relationship between system accuracy and the time required for final location estimation, we modeled the TSCH network scenario that is available by default in the Contiki-NG operating system. A slotframe with eight timeslots is used with one dedicated cell for packet exchange between devices. With a modified timeslot prolonged to 15 ms, the root can exchange a message with its neighboring node every 120 ms. When the median of 16 estimates is taken as a final result, the location acquisition time equals 1920 ms. As a trade-off between update time and accuracy, we propose to obtain five successive measurements, where the root requires 600 ms for a final location estimate. As shown in Figure 4b, the maximum localization error of the system remains below 2 m, and the average error is 0.37 m.

**Figure 5:**
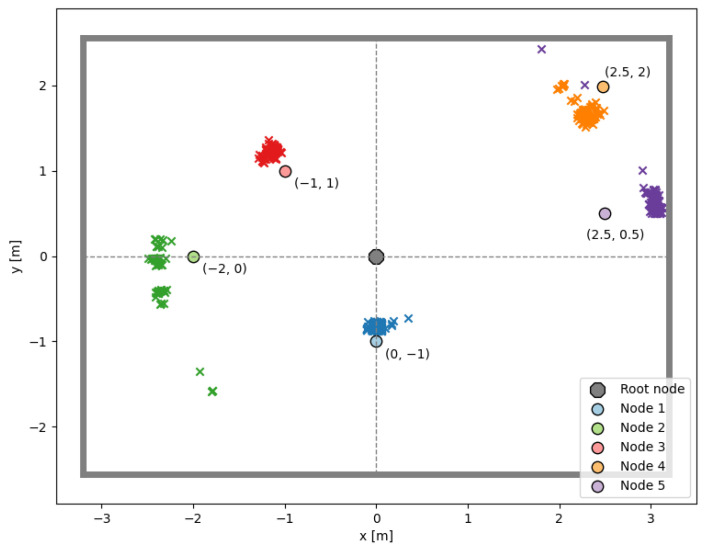
Actual (circles) and estimated (crosses) locations of devices in an office room based on five successive measurements as a final result.

Detailed distance and angle estimation results with a median of five successive measurements are presented in Table 4. The median (MED) error helps to understand the typical error magnitude or accuracy while minimizing the impact of outliers. The standard deviation (STD) provides an understanding of the variability of the estimations around their MED, indicating the precision of estimations. The MAE assesses the average absolute error in the estimations, providing insights into systematic biases. Relatively large errors in the location estimates can be attributed to the inaccurate angle estimations, which can also be observed in Figure 5. Improved angle estimation results can be achieved with a larger number of antenna elements in the UCA and a spatial smoothing technique. In addition, along with tuning of individual dipole antennas to the 2.4 GHz band, the effects of mutual coupling should be considered.

The STD of the measured distances is relatively small, which proves the precision of the MCPD distance estimation algorithm, especially after removing the outliers with the median of five successive measurements. The MAE is below 0.6 m with only 15 phase samples, which is comparable to PBR approaches that collected 160 phase samples [22]. The MAE of the estimated distances increases as the devices are positioned closer to the walls due to multipath effects. For example, Nodes 1 and 3, which are only 1 m and 1.5 m away from the root, respectively, show better distance estimation accuracy than other nodes. Algorithms that are less susceptible to noise and multipath effects can be used to improve the accuracy of the estimated distances. In addition, consecutive distance estimations can be made with different antenna elements to exploit the antenna diversity.

The IEEE 802.15.4 standard currently supports RSSI measurement during communication, which can be used to approximate the distance to the source of the transmission. The relationship between RSSI and distance can be modeled using the common free-space path loss (FSPL) formula [60], which describes how the radio signal degrades as it travels through space. This baseline distance estimation solution is compared with the proposed MCPD approach by analyzing an ideal scenario, where the fluctuations in the RSSI due to multipath propagation effects and noise are neglected, and the RSSI is measured with a granularity of 1 dBm [8]. From the FSPL equation, we derive how the small changes in RSSI measurement ΔRSSI affect the distance estimation error Δd using

(1) $$\Delta d=d\times \frac{\Delta RSSI\ln10}{20}.$$

A 1 dBm change in the RSSI value results in an approximately 11.5% change in the calculated distance. For example, at the distance of 1 m, the estimation error is within 0.115 m, while for distances above 10m, the estimation error can exceed 1 m. By increasing the distance between the receiver and transmitter, even small fluctuations in RSSI readings lead to high variability in the calculated distance, while variations in the MCPD distance estimation remain low even with the increased distance between the devices [23].

## 7. Conclusions

In this paper, we propose a hybrid localization approach based on distance and angle estimates integrated with the TSCH protocol, enabling localization functionality alongside communication. The phase measurement process (PMP) is designed, and its integration into the protocol is described. Each packet transmitted includes both DoA and MCPD distance estimates that can be used to localize an unknown device. The channel hopping functionality of the protocol and several successive measurements were considered to mitigate multipath effects and remove outliers. The proposed method was validated based on a measurement campaign in an office environment utilizing low-cost proprietary devices. The results show a few tens of centimeters of accuracy in the investigated indoor scenario. In future work, the presented solution will be tested in various environments, and its scalability will be analyzed by designing a dedicated SF. Additionally, the solution will be extended with antenna diversity for distance estimation, spatial smoothing for DoA estimation, and different spectrum-peak search algorithms to improve performance. With the proposed advancements, we assume that new possibilities will be offered for sensing and localization applications in indoor environments.

## Figures and Tables

**Table 2:** Number of obtained estimations and number of discarded estimations at each node with the use of Quality Indicator (QI).

	No. of Estimates	No. of Discarded
Node 1	651	6
Node 2	307	21
Node 3	245	0
Node 4	604	161
Node 5	571	3

**Table 3:** Estimated location errors with different numbers of successive measurements taken as the final result.

	Single		Successive 5		Successive 16	
	RMSE	MAE	RMSE	MAE	RMSE	MAE
Node 1	0.374	0.171	0.194	0.112	0.185	0.101
Node 2	2.178	0.824	0.611	0.333	0.452	0.287
Node 3	0.349	0.189	0.278	0.185	0.268	0.183
Node 4	2.506	0.956	0.410	0.267	0.390	0.265
Node 5	1.068	0.486	0.628	0.361	0.583	0.349

**Table 4:** Detailed distance and angle estimation results per node. A median of five successive measurements has been used as the final estimation result.

	True Distance	Distance Estimates [m]			True Angle	Angle Estimates [°]		
	[m]	MED	STD	MAE	[°]	MED	STD	MAE
Node 1	1.000	0.818	0.035	0.179	270	272.0	4.10	3.01
Node 2	2.000	2.399	0.041	0.395	180	181.1	10.75	7.47
Node 3	1.500	1.678	0.044	0.178	135	133.8	2.35	2.45
Node 4	3.200	2.836	0.060	0.362	38.6	36.3	2.70	3.09
Node 5	2.540	3.133	0.036	0.587	11.3	11.5	5.03	1.90

## Data Availability

The driver for the AT86RF215 radio within the Contiki-NG operating system is available online in [59].

## Abbreviations

The following abbreviations are used in this manuscript:

2D	two-dimensional
AR	Active Reflector
BLE	Bluetooth Low Energy
CDF	Cumulative Distribution Function
CFO	Carrier Frequency Offset
CIR	Channel Impulse Response
CSI	Channel State Information
CW	Continuous Wave
DAC	Digital-to-Analog Converter
DoA	Direction of Arrival
DODAG	Destination-Oriented Directed Acyclic Graph
ESPRIT	Estimation of Signal Parameters via Rotational Invariance Technique
FFT	Fast Fourier Transform
FSPL	free-space path loss
GFSK	Gaussian Frequency Shift Keying
iCDE	interpolated Complex-valued Distance Estimation
ILAC	Integrated Localization and Communication
IoT	Internet of Things
IQ	In-Phase and Quadrature
ISAC	Integrated Sensing and Communication
MAC	Medium Access Layer
MAE	Mean Absolute Error
MCPD	Multi-Carrier Phase Difference
MED	Median
ML	Machine Learning
MUSIC	MUltiple SIgnal Classification
OFDM	Orthogonal Frequency-Division Multiplexing
O-QPSK	Offset Quadrature Phase Shift Keying
PBR	Phase-Based Ranging
PHY	Physical Layer
PLL	Phase-Locked Loop
PMP	Phase Measurement Process
PMU	Phase Measurement Unit
PPM	Pulse Position Modulation
QF	Quality Factor
QI	Quality Indicator
RF	Radio Frequency
RMSE	Root Mean Square Error
RPL	Routing Protocol for Low-Power and Lossy Networks
RSSI	Received Signal Strength Indicator
SPI	Serial Peripheral Interface
STD	Standard Deviation
ToA	Time of Arrival
TSCH	Time-Slotted Channel Hopping
UCA	Uniform Circular Array
UR	Unambiguous Range
UWB	Ultra-Wideband
